# Meta-QTL Analysis Reveals Consensus Genomic Regions and Candidate Genes for Resistance to Sudden Death Syndrome in Soybean

**DOI:** 10.3390/plants15172691

**Published:** 2026-09-02

**Authors:** Stella K. Kantartzi

**Affiliations:** Plant Genetic Improvement Lab, School of Agricultural Sciences, Southern Illinois University, Carbondale, IL 62901, USA; stella.kantartzi@siu.edu

**Keywords:** *Glycine max*, *Fusarium virguliforme*, quantitative trait loci, genome-wide association study, candidate genes, marker-assisted selection, disease resistance, salicylic acid signaling

## Abstract

Sudden death syndrome (SDS), caused by *Fusarium virguliforme*, is one of the most economically important diseases limiting soybean production worldwide. Although numerous quantitative trait loci (QTL) associated with SDS resistance have been reported, inconsistencies among mapping populations, marker systems, and experimental conditions have hindered the identification of robust resistance loci for soybean improvement. In this study, a comprehensive meta-analysis was conducted to integrate published QTL and identify stable consensus genomic regions associated with SDS resistance. After a systematic literature survey and data curation, 153 QTL derived from 14 linkage-mapping studies were analyzed using a custom R-based workflow, resulting in the identification of 23 consensus meta-QTL (MQTL) distributed across 17 chromosomes. Several MQTL, particularly those located on chromosomes 6, 8, 18, and 20, were supported by multiple independent studies and represented major genomic hotspots for SDS resistance. Physical localization and functional annotation of these MQTL identified 217 candidate genes, including genes predicted to be involved in plant defense, signal transduction, transcriptional regulation, and secondary metabolism. Gene Ontology enrichment analysis identified response to salicylic acid as the only biological process that remained significant after FDR correction, whereas Kyoto Encyclopedia of Genes and Genomes pathway analysis did not identify significantly enriched pathways. Independent support using five published genome-wide association studies further supported several MQTL, especially those on chromosomes 6, 18, and 20, thereby increasing confidence in these genomic regions. The identified MQTL and prioritized candidate genes provide potential genomic resources for future marker development, improvement applications, and functional validation aimed at improving soybean resistance to SDS.

## 1. Introduction

Soybean (*Glycine max* (L.) Merr.) is one of the world’s most important legume crops, providing a major source of plant protein and oil for human consumption, livestock feed, and many industrial applications [1]. Global soybean production continues to increase in response to growing demand; however, obtaining stable yields remains challenging due to biotic and abiotic stresses that limit productivity [2]. Among the most economically important diseases affecting soybean production is sudden death syndrome (SDS), a destructive root and foliar disease caused by *Fusarium virguliforme* [3]. Since its initial description in the United States, SDS has become one of the most damaging soybean diseases in North and South America, causing substantial yield losses under favorable conditions for disease development [4]. Disease severity is affected by complicated interactions among pathogen virulence, environmental conditions, and host genotype, making the development of resistant cultivars the most durable and ecologically friendly strategy for disease management [5].

Resistance to SDS is a complex quantitative trait controlled by multiple loci with small-to-moderate genetic effects [6]. During the past three decades, numerous linkage mapping studies have identified quantitative trait loci (QTL) associated with SDS resistance using diverse biparental populations, molecular marker platforms, and phenotyping protocols [7]. These studies have substantially improved our understanding of the genetic architecture underlying resistance and have identified genomic regions distributed across multiple soybean chromosomes. However, direct comparison of reported QTL remains challenging because differences in mapping populations, marker density, phenotyping protocols, environmental conditions, and statistical approaches often result in inconsistent QTL positions and confidence intervals [6,7]. Consequently, many reported QTL cannot be readily translated into practical tools for marker-assisted selection or incorporated into soybean improvement programs.

Meta-analysis of QTL (MQTL) provides an effective strategy for integrating results from independent genetic studies and identifying consensus genomic regions that are consistently associated with a target trait [8]. By combining information across multiple experiments, consensus MQTL reduce redundancy among overlapping QTL, refine genomic intervals, and improve confidence in the localization of resistance loci [9,10]. The resulting consensus regions provide more reliable targets for marker-assisted selection, genomic selection, and functional characterization [11,12].

The increasing availability of high-quality soybean genome assemblies and publicly accessible genomic databases has further enhanced the value of MQTL [13]. Physical localization of consensus regions enables the systematic identification of genes located near resistance-associated loci, while functional annotation provides insight into the biological mechanisms underlying disease resistance [12]. In addition, comparison of MQTL with independent genome-wide association studies (GWAS) provides independent support by determining whether consensus regions identified from linkage mapping coincide with loci detected in diverse association panels [14]. Therefore, integrating evidence from linkage mapping, candidate gene analysis, and GWAS provides a more comprehensive framework for prioritizing genomic regions and genes associated with SDS resistance.

Although numerous QTL associated with SDS resistance have been reported, a comprehensive synthesis integrating published linkage mapping studies, candidate gene identification, and GWAS support has not previously been available. Differences in mapping populations, marker systems, genetic map density, phenotyping protocols, environmental conditions, and statistical methodologies complicate direct comparisons among studies and often result in inconsistent QTL positions. Such heterogeneity among studies represents an inherent limitation when integrating published QTL datasets. Although systematic data curation and standardization can improve comparability among studies, differences in mapping populations, phenotyping systems, environments, marker platforms, and statistical approaches cannot be eliminated and therefore represent an inherent limitation of QTL integration [7].

To address these challenges, published linkage mapping studies were integrated using a custom meta-analysis workflow, and the resulting consensus MQTL were combined with candidate gene identification, functional enrichment analysis, and independent GWAS support. Therefore, the objectives of this study were to (i) systematically compile and curate published QTL associated with SDS resistance, (ii) identify consensus MQTL using a custom R-based workflow, (iii) physically localize MQTL and identify candidate genes within their genomic regions, (iv) characterize these genes through functional annotation and enrichment analyses, and (v) evaluate MQTL using evidence from previously published soybean GWAS. Overall, these analyses provide a comprehensive genomic framework for understanding the genetic basis of SDS resistance and facilitate the development of molecular tools for soybean improvement.

## 2. Results

### 2.1. Literature Survey and Characteristics of the Curated QTL Dataset

**Figure 1:**
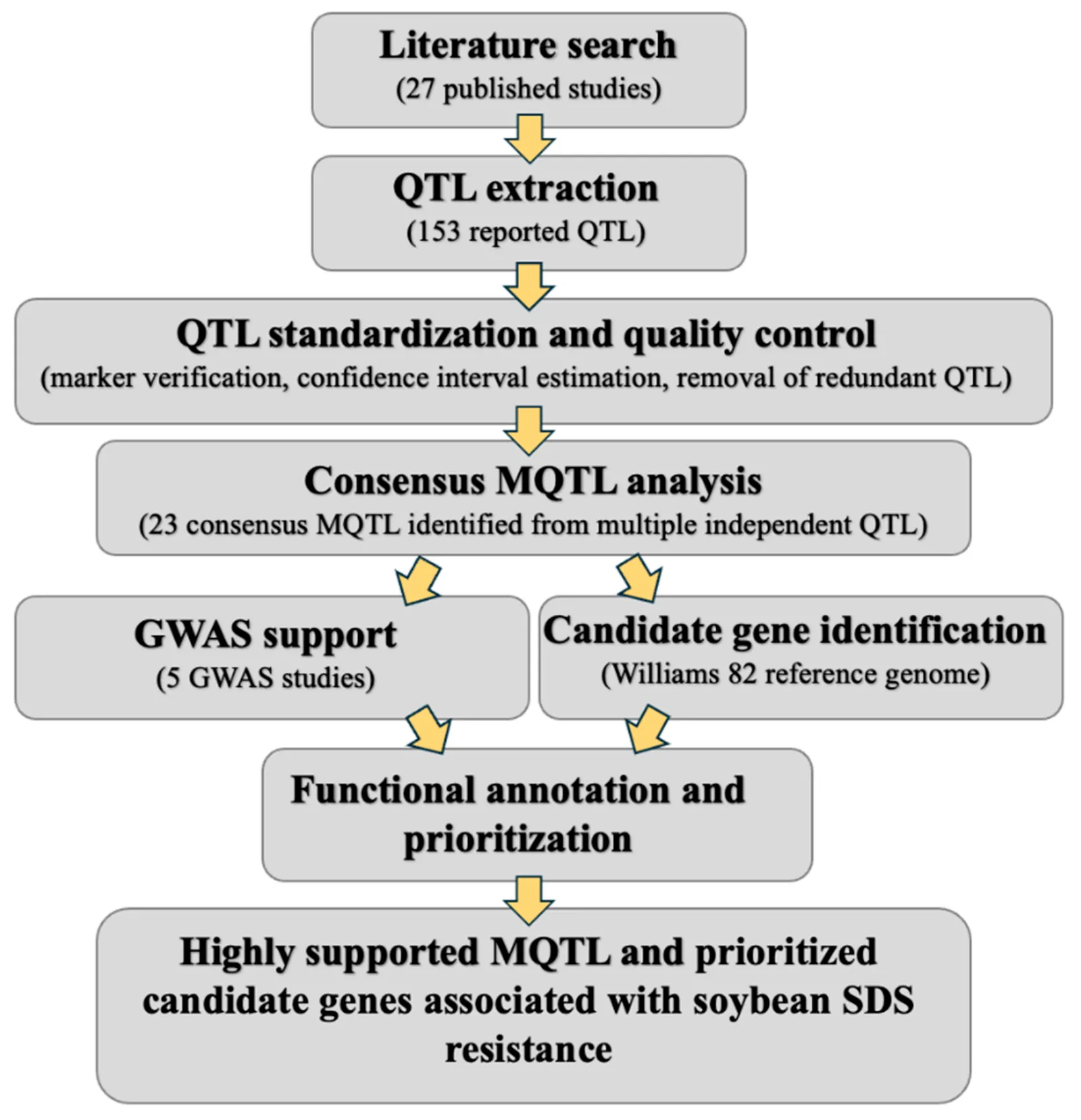
Workflow of the meta-analysis conducted to identify consensus meta-QTL (MQTL) associated with sudden death syndrome (SDS) resistance in soybean. Published QTL studies were systematically reviewed, and QTL from 14 eligible linkage mapping studies were extracted, standardized, and curated prior to MQTL analysis. Consensus MQTL were subsequently compared with GWAS and integrated with SoyBase annotations to identify and prioritize candidate genes associated with SDS resistance.

**Figure 2:**
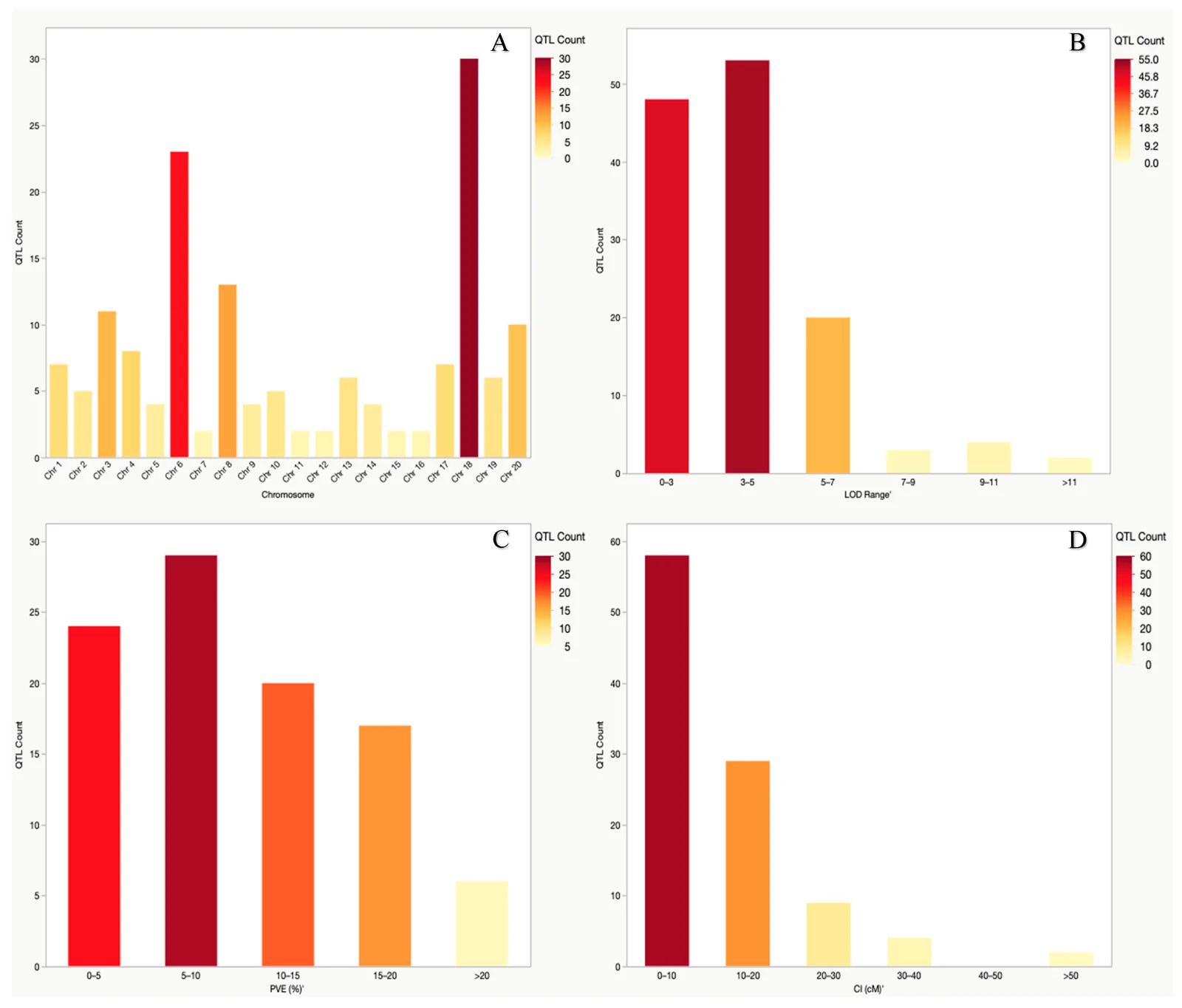
Characteristics of the curated QTL dataset used for meta-analysis. (**A**) Genome-wide distribution of the 153 curated QTL across the 20 soybean chromosomes. (**B**) Distribution of the maximum phenotypic variance explained (PVE) by the curated QTL. (**C**) Distribution of QTL confidence intervals (CI). (**D**) Distribution of QTL support based on the number of independent studies reporting overlapping genomic regions.

The overall workflow of the meta-analysis is presented in Figure 1. A comprehensive literature survey identified 27 independent studies published between 1996 and 2022 that investigated the genetic architecture of SDS resistance in soybean. Following quality assessment and data curation, 14 studies containing sufficient mapping information for consensus analysis were retained for meta-analysis (Table 1). Complete information for all studies, including mapping populations, phenotypic traits, marker types, and original QTL information, is provided in Table S1. Following marker verification, confidence interval estimation, standardization of genomic positions, and removal of redundant or incomplete records, a curated dataset comprising 153 QTL distributed across all 20 soybean chromosomes was assembled for meta-analysis (Table S2). The chromosomal distribution, phenotypic variance explained, confidence interval distribution, and supporting evidence of the curated QTL are summarized in Figure 2.

### 2.2. Identification of Consensus MQTL

**Figure 3:**
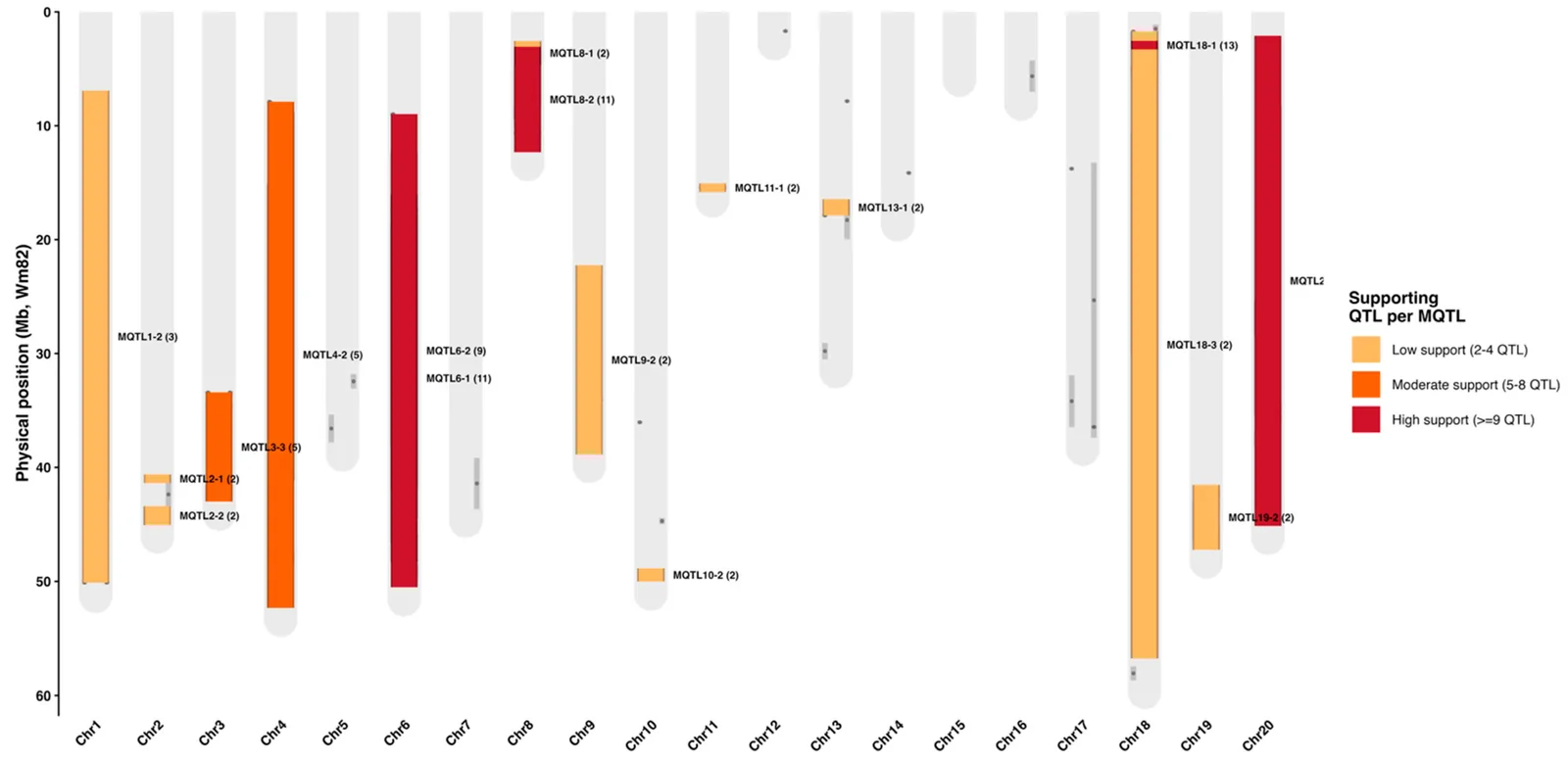
Genome-wide distribution of the 23 consensus meta-QTL (MQTL) according to their physical positions on the Williams 82 reference genome. Colored bars represent the physical intervals of individual MQTL, whereas the gray chromosome ideograms indicate chromosome lengths. MQTL are color-coded according to the number of supporting original QTL: low support (2–4 QTL; light orange), moderate support (5–8 QTL; orange), and high support (≥9 QTL; dark red). Numbers in parentheses following each MQTL identifier indicate the number of original QTL integrated into that consensus region.

**Figure 4:**
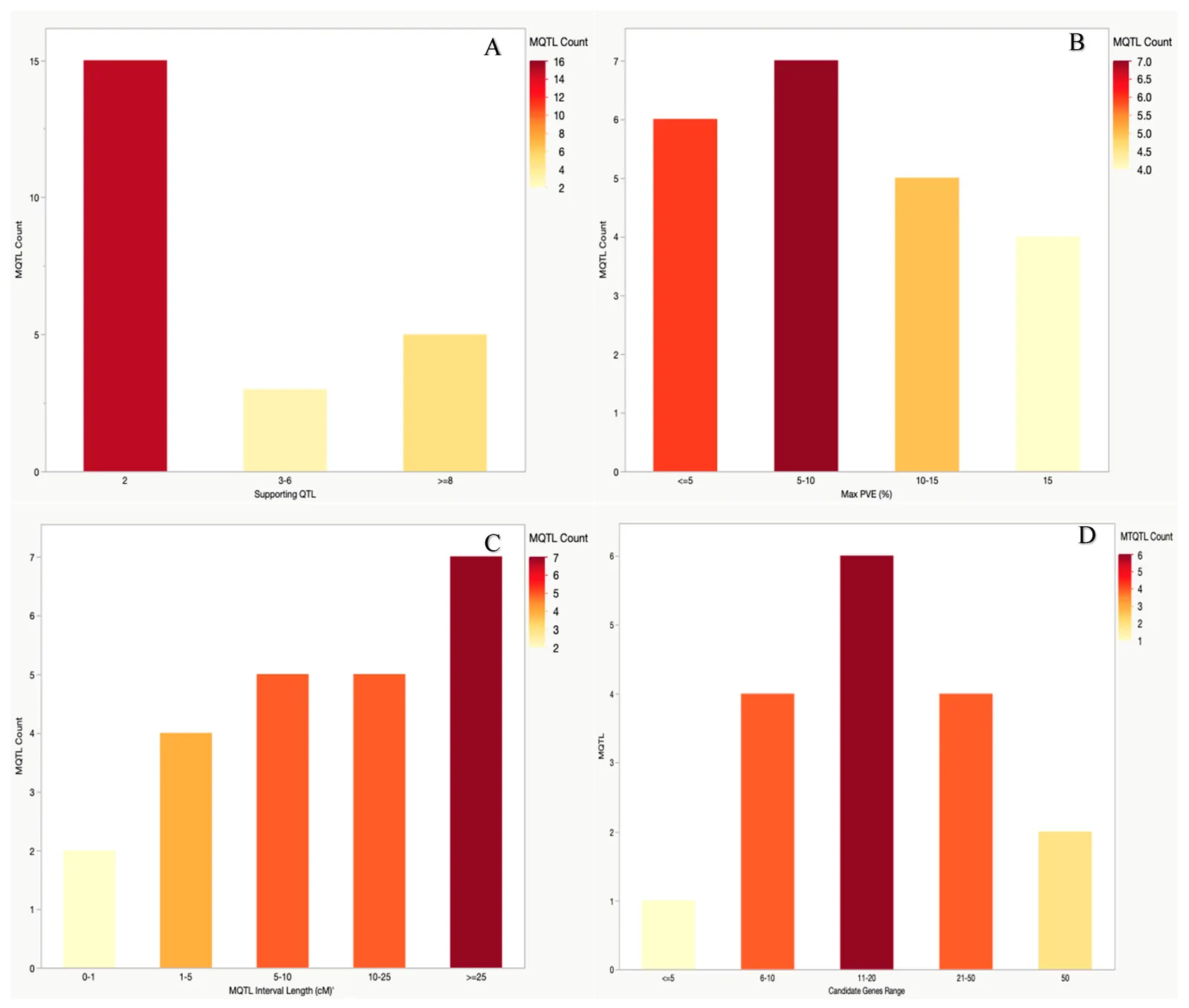
Characteristics of the 23 consensus meta-QTL (MQTL). (**A**) Distribution of MQTL according to the number of supporting QTL. (**B**) Distribution of the maximum phenotypic variance explained (PVE) by the identified MQTL. (**C**) Distribution of MQTL confidence limits. (**D**) Distribution of the number of candidate genes identified within MQTL regions.

Consensus analysis identified 23 MQTL distributed across 17 soybean chromosomes (1, 2, 3, 4, 5, 6, 8, 9, 10, 11, 13, 14, 15, 17, 18, 19, and 20), indicating that multiple independently reported QTL converged on common genomic regions (Table 2). MQTL were unevenly distributed across the genome, with chromosomes 6, 18, and 20 containing the most strongly supported consensus regions, whereas chromosomes 1, 6, 8, and 18 each harbored multiple MQTL (Figure 3). The number of supporting QTL per MQTL ranged from two to 13. Of the 23 identified MQTL, 15 were supported by two original QTL, three by three to six QTL, and five by eight or more QTL (Figure 4A). MQTL18-1 represented the strongest consensus region, integrating 13 original QTL, followed by MQTL6-1 (11 QTL), MQTL8-2 (11 QTL), MQTL6-2 (9 QTL), and MQTL20-1 (9 QTL), which also represented major genomic hotspots for SDS resistance. Maximum phenotypic variance explained (PVE) among the identified MQTL ranged from 0.04% to 33.3%. Among the 22 MQTL with available PVE information, nine exhibited maximum PVE values ≥10%, whereas seven, one, and five MQTL fell within the 5–10%, 2–5%, and 0–2% categories, respectively (Figure 4B). One MQTL lacked PVE information and therefore could not be classified. MQTL confidence intervals ranged from 0.12 to 110.8 cM, with most consensus regions falling within the 10–25 cM and ≥25 cM interval classes (Figure 4C). Overall, integration of overlapping QTL reduced redundancy among previously reported loci and identified shared consensus genomic regions, although the width of individual MQTL intervals varied considerably.

### 2.3. Candidate Gene Identification and Functional Characterization

A total of 217 candidate genes were identified within the physical intervals of the 23 MQTL using a ±50 kb window surrounding each consensus region. The number of candidate genes per MQTL ranged from 4 to 53, with most MQTL containing between 11 and 20 genes (Figure 4D). MQTL6-2 and MQTL18-3 contained the largest numbers of candidate genes, whereas MQTL11-1 contained the lowest numbers. Functional annotation revealed that the candidate genes are associated with diverse biological processes related to plant defense and stress responses. Major functional categories included receptor-like kinases, nucleotide-binding leucine-rich repeat (NLR) proteins, transcription factors, protein kinases, transporters, hormone signaling components, oxidative stress-related proteins, enzymes involved in cell wall organization and phenylpropanoid metabolism, and proteins associated with secondary metabolism. Several candidate genes encoded proteins previously implicated in plant immune responses, including leucine-rich repeat receptor-like kinases, mitogen-activated protein kinase-related proteins, WRKY and MYB transcription factors, cytochrome P450 enzymes, glutathione S-transferases, UDP-glycosyltransferases, ATP-binding cassette transporters, and disease resistance proteins (Table 3).

### 2.4. Independent Support of Consensus MQTL Using Published GWAS

**Figure 5:**
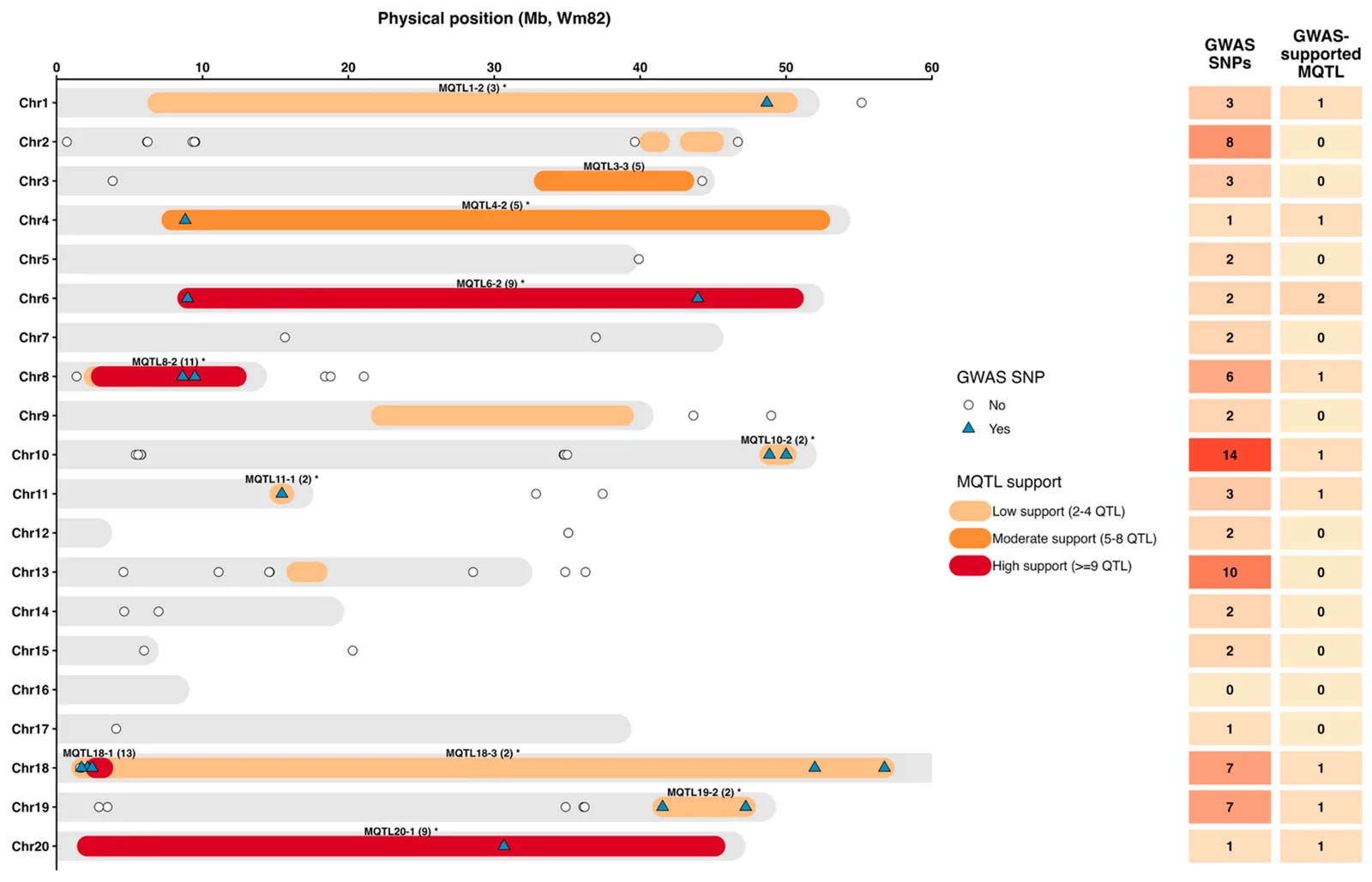
Independent support of consensus meta-QTL (MQTL) using previously reported genome-wide association studies (GWAS). Consensus MQTL are displayed according to their physical positions on the Williams 82 soybean reference genome. Colored bars represent MQTL intervals and are classified according to the number of supporting QTL: low support (2–4 QTL; light orange), moderate support (5–8 QTL; orange), and high support (≥9 QTL; dark red). Open circles indicate published GWAS SNPs, whereas blue triangles denote GWAS SNPs that colocalize with MQTL intervals. Numbers in parentheses following each MQTL identifier indicate the number of original QTL integrated into each consensus region. The heat map summarizes the number of GWAS SNPs identified on each chromosome and the number of GWAS-supported MQTL. MQTL supported by independent GWAS evidence are indicated by an asterisk (*).

To independently evaluate the identified MQTL, significant single-nucleotide polymorphisms (SNPs) reported in five previously published GWAS for SDS resistance were compared with the physical positions of the identified MQTL (Table 4). Physical colocalization between MQTL and GWAS loci was assessed using the Williams 82 soybean reference genome (Wm82.a2.v1). Physical colocalization analysis identified 17 significant GWAS SNPs within 10 of the 23 consensus MQTL: MQTL1-2, MQTL4-2, MQTL6-1, MQTL6-2, MQTL8-2, MQTL10-2, MQTL11-1, MQTL18-3, MQTL19-2, and MQTL20-1. The strongest concordance between linkage mapping and association mapping was observed for MQTL located on chromosomes 6, 18, and 20, where multiple significant SNPs colocalized with highly supported MQTL identified through the meta-analysis. These regions were also supported by multiple independent linkage mapping studies, reinforcing their importance as stable genomic regions associated with SDS resistance. The genome-wide relationship between MQTL and published GWAS loci is illustrated in Figure 5, where significant GWAS SNPs are displayed relative to the physical positions of the identified MQTL. Integration of linkage mapping and GWAS evidence provided additional support for several consensus regions and facilitated the prioritization of candidate genes for downstream biological investigation.

### 2.5. Functional Enrichment Analysis of MQTL Candidate Genes

**Figure 6:**
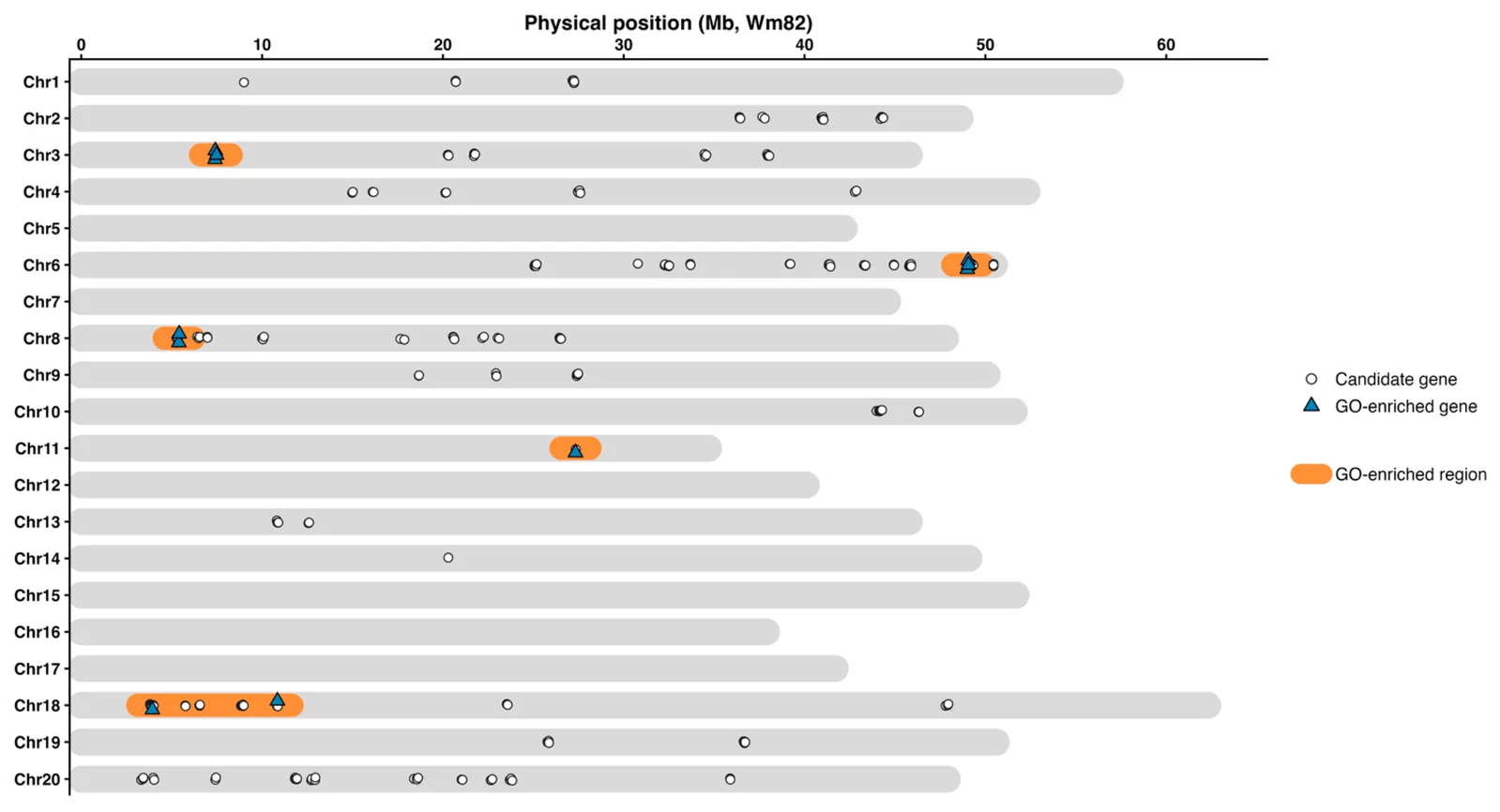
Genome-wide distribution of GO-enriched candidate genes identified within consensus meta-QTL (MQTL). Gray bars represent the physical lengths of the 20 soybean chromosomes based on the Williams 82 reference genome. Open circles indicate candidate genes identified within MQTL intervals, blue triangles denote candidate genes associated with significantly enriched Gene Ontology (GO) biological processes, and orange bars indicate genomic regions containing clusters of GO-enriched candidate genes.

Gene Ontology (GO) enrichment analysis was performed using the complete non-redundant set of 217 candidate genes identified within the 23 MQTL. GO-enriched genes were concentrated within MQTL located on chromosomes 3, 6, 8, 11, and 18 (Figure 6). Response to salicylic acid (GO:0009751) was the only biological process that remained significantly enriched after false discovery rate correction (FDR = 0.0299). Additional enriched biological processes included regulation of salicylic acid-mediated signaling pathway, salicylic acid-mediated signaling pathway, cellular response to salicylic acid stimulus, phenylpropanoid metabolic process, phenylpropanoid catabolic process, lignin catabolic process, and secondary metabolic process. Collectively, these results indicate that the identified candidate genes are primarily associated with defense signaling and secondary metabolism. In contrast, Kyoto Encyclopedia of Genes and Genomes (KEGG) pathway analysis did not identify significantly enriched pathways after FDR correction, suggesting that the candidate genes are distributed across diverse biological pathways rather than concentrated within a single metabolic pathway.

## 3. Discussion

### 3.1. Overview of the Major Findings

This study provides a comprehensive synthesis of the genetic architecture underlying soybean resistance to SDS by integrating published QTL, consensus MQTL, candidate gene identification, functional enrichment analysis, and independent GWAS support. Using a custom R-based meta-analysis workflow, 153 curated QTL were consolidated into 23 consensus MQTL distributed across 17 soybean chromosomes, substantially reducing redundancy among previously reported loci and identifying consensus genomic regions associated with SDS resistance. Several MQTL, particularly MQTL18-1, MQTL6-1, MQTL6-2, MQTL8-2, and MQTL20-1, were supported by multiple independent studies, indicating that these regions represent stable components of the genetic architecture underlying SDS resistance. Candidate gene analysis identified numerous genes associated with plant immunity, including receptor-like kinases, NLR proteins, transcription factors, protein kinases, transporters, and enzymes involved in secondary metabolism. Functional enrichment analysis further highlighted the importance of salicylic acid-related biological processes, whereas independent GWAS support strengthened confidence in several consensus regions, particularly those located on chromosomes 6, 18, and 20. These findings demonstrate that integrating MQTL analysis with candidate gene prioritization and GWAS support provides a framework for identifying well-supported resistance loci and candidate genes for marker-assisted selection, genomic selection, and future functional validation in soybean.

### 3.2. Meta-Analysis Improves the Resolution and Reliability of SDS Resistance Loci

Resistance to SDS is a quantitatively inherited trait controlled by numerous loci with varying genetic effects and environmental stability [6,7]. Consequently, individual QTL studies often report inconsistent genomic regions because of differences in mapping populations, marker density, disease evaluation methods, environmental conditions, and statistical approaches [6,7]. Meta-analysis addresses these limitations by integrating independent QTL studies into consensus genomic regions, increasing confidence in loci that are consistently detected across diverse genetic backgrounds [9]. This approach has been successfully applied to several complex traits in soybean, including seed composition, seed quality, agronomic traits, and disease resistance, demonstrating its ability to refine QTL positions and identify targets for crop improvement [10,11,12,13]. In the present study, integration of 153 curated QTL into 23 consensus MQTL substantially simplified the published landscape of SDS resistance loci. Several MQTL, particularly MQTL18-1, MQTL6-1, MQTL6-2, MQTL8-2, and MQTL20-1, were supported by multiple independent QTL originating from different mapping populations, indicating that these genomic regions are repeatedly associated with SDS resistance across independent studies. The repeated detection of these regions suggests that they harbor loci contributing to SDS resistance across diverse genetic backgrounds and experimental conditions. The consolidation of overlapping QTL into consensus genomic intervals also facilitates downstream candidate gene discovery. Individual QTL often span large genomic regions containing numerous genes, making the identification of causal genes challenging. By reducing redundancy and integrating evidence from multiple studies, MQTL analysis provides consensus genomic targets for functional characterization, fine mapping, and marker development [9,10,11,12,13]. This prioritization can improve the efficiency of identifying biologically relevant genes and selecting candidates for experimental validation.

The identification of highly supported MQTL on chromosomes 6, 8, 18, and 20 is consistent with previous linkage mapping studies that repeatedly detected SDS resistance loci in these genomic regions [6,7]. Rather than identifying entirely novel loci, the present study consolidates nearly three decades of independent genetic evidence into a set of consensus MQTL, providing a comprehensive genomic framework for future genetic studies and soybean improvement programs.

### 3.3. Biological Significance of Candidate Genes and Enriched Defense Pathways

The integration of consensus MQTL with candidate gene identification and functional enrichment analysis provided biological support for the genomic regions associated with SDS resistance. Candidate genes identified within MQTL intervals encoded proteins involved in pathogen perception, signal transduction, transcriptional regulation, secondary metabolism, and stress responses, indicating that resistance is controlled by a coordinated defense network rather than by a single resistance gene [15,16,17,18]. The concentration of defense-related genes within several highly supported MQTL further strengthens the biological relevance of these consensus regions. Among the enriched biological processes, salicylic acid-mediated signaling emerged as the most significant. Salicylic acid is a central regulator of systemic acquired resistance and plays a fundamental role in activating plant immune responses against biotrophic and hemi-biotrophic pathogens [19,20]. Although *F. virguliforme* is generally regarded as a necrotrophic pathogen during symptom development, increasing evidence indicates that salicylic acid signaling also contributes to limiting early pathogen colonization and activating downstream defense responses in soybean [21,22]. The enrichment of genes associated with salicylic acid signaling suggests that this pathway represents an important component of the molecular mechanisms underlying SDS resistance. Functional enrichment analysis also highlighted genes involved in phenylpropanoid metabolism, lignin catabolism, and secondary metabolism. The phenylpropanoid pathway produces lignin, flavonoids, and numerous antimicrobial compounds that reinforce cell walls and restrict pathogen invasion [23,24]. Enhanced lignification and phenolic compound accumulation have been associated with increased resistance to fungal pathogens, including *F. virguliforme* [25]. The enrichment of these biological processes suggests that structural reinforcement and secondary metabolite production contribute to quantitative resistance against SDS. Beyond the significant GO term, additional biological processes related to phenylpropanoid metabolism, lignin catabolism, and secondary metabolism were represented among the candidate genes but did not remain significant after FDR correction. These patterns should therefore be considered suggestive rather than statistically supported enrichment. Similarly, no KEGG pathway reached statistical significance after FDR correction, indicating that pathway-level interpretations should also be considered exploratory. Several candidate genes encoded receptor-like kinases, NLR proteins, protein kinases, WRKY transcription factors, cytochrome P450 enzymes, transport proteins, and enzymes involved in oxidative stress regulation. These gene families constitute core components of the plant innate immune system by mediating pathogen recognition, intracellular signal transduction, transcriptional reprogramming, reactive oxygen species homeostasis, and activation of downstream defense responses [17,18,26,27,28]. Their repeated occurrence within multiple MQTL suggests that SDS resistance is controlled by interconnected defense pathways rather than isolated genetic factors. Candidate gene prioritization was based primarily on functional annotation; therefore, expression, sequence variation, and functional studies will be required to validate the roles of these genes in SDS resistance. Taken together, the candidate gene and functional enrichment analyses provide biological context for the consensus MQTL identified in this study. These genes represent promising targets for further evaluation through gene expression analyses, fine mapping, genome editing, and reverse genetics, which will help determine their potential utility for improving soybean resistance to SDS.

### 3.4. Independent GWAS Evidence Supports the Identified MQTL

Independent support of consensus MQTL using GWAS provides an additional level of evidence regarding the reliability of genomic regions identified through meta-analysis. Although linkage mapping detects loci segregating within biparental populations, GWAS exploits historical recombination across genetically diverse germplasm, often providing higher mapping resolution [29,30]. Consequently, genomic regions identified by both approaches represent well-supported loci for further investigation. Comparison of published GWAS with the consensus MQTL identified in this study demonstrated that several significant association signals colocalized with MQTL intervals. MQTL located on chromosomes 1, 4, 6, 8, 10, 11, 18, 19, and 20 were supported by independent GWAS evidence, indicating that these genomic regions have been repeatedly associated with SDS resistance using complementary genetic approaches. The strongest concordance was observed for MQTL6-2, which was supported by two independent GWAS SNPs, whereas several additional MQTL were supported by one or more significant association signals. The convergence of linkage mapping and association mapping substantially increases confidence that these genomic regions contain loci contributing to quantitative resistance. Despite the increasing use of GWAS to investigate soybean disease resistance, relatively few association signals overlapped with the MQTL identified in the present study. This limited overlap likely reflects differences in germplasm composition, marker density, phenotyping protocols, statistical models, allele frequencies, and linkage disequilibrium among individual studies [29,30]. Furthermore, linkage mapping and GWAS capture complementary components of the genetic architecture of complex traits, making complete concordance unlikely [29,30]. Nevertheless, the repeated identification of several consensus regions by both approaches demonstrates that these MQTL represent consistently supported genomic regions associated with SDS resistance that have persisted across diverse genetic backgrounds and experimental conditions. The integration of MQTL and GWAS provides a valuable framework for prioritizing genomic regions for future research. MQTL supported by independent GWAS evidence constitute well-supported targets for fine mapping, candidate gene validation, marker-assisted selection, genomic selection, and functional genomics. These GWAS-supported consensus regions therefore represent promising targets for identifying the causal genes underlying soybean resistance to SDS.

### 3.5. Implications for Soybean Improvement and Future Perspectives

The consensus MQTL identified in this study provide valuable genomic resources for improving soybean resistance to SDS. Compared with individual QTL reported in isolated linkage mapping studies, consensus MQTL represent genomic regions supported by multiple independent experiments, making them more reliable targets for marker-assisted and genomic selection [31,32]. In particular, the highly supported MQTL on chromosomes 6, 8, 18, and 20, together with regions independently supported by published GWAS, constitute well-supported loci that can be prioritized for molecular marker development and soybean improvement. By integrating linkage mapping, GWAS, candidate gene identification, and functional annotation, this study provides a comprehensive framework for prioritizing resistance loci and accelerating genetic improvement for SDS resistance. Despite these strengths, several limitations should be acknowledged. The present meta-analysis relied on previously published QTL generated from diverse mapping populations, marker systems, disease evaluation methods, and environmental conditions. Although the data were carefully curated and standardized before analysis, methodological differences among studies may have influenced the estimated positions and confidence intervals of some MQTL. In addition, the provisional ±1 cM interval assigned to QTL lacking reported confidence intervals or sufficient flanking-marker information represents a methodological assumption that may have influenced interval-overlap clustering and the resulting MQTL boundaries. Furthermore, candidate gene identification was based primarily on genomic colocalization and functional annotation; therefore, the biological functions of the prioritized genes remain to be experimentally validated through approaches such as transcriptome profiling, fine mapping, genome editing, and reverse genetics. Future studies should integrate high-resolution mapping populations with transcriptomics, proteomics, metabolomics, and functional genomics to identify the causal genes underlying the most promising MQTL [33]. Incorporating validated MQTL into genomic prediction models, marker-assisted selection, and genome editing strategies has the potential to accelerate the development of soybean cultivars with durable SDS resistance while maintaining high yield potential and agronomic performance [31,32,33]. As additional genetic mapping studies become available, periodic updates of MQTL analyses will further refine consensus regions and improve the identification of candidate genes associated with soybean disease resistance.

## 4. Materials and Methods

### 4.1. Literature Search and Data Collection

A comprehensive literature search was conducted to identify published studies investigating the genetic basis of soybean SDS resistance. Searches were performed using Google Scholar, Web of Science, Scopus, and SoyBase to maximize coverage of peer-reviewed publications and publicly available genomic resources. The search included studies published through May 2026 using combinations of the keywords *soybean*, *sudden death syndrome*, *Fusarium virguliforme*, *QTL*, *quantitative trait loci*, *genetic mapping*, *linkage mapping*, *molecular markers*, *genome-wide association study*, *GWAS*, and *disease resistance*. The literature survey identified 27 independent studies published between 1996 and 2022, although the literature search itself included publications available through May 2026. Only linkage mapping studies reporting QTL in biparental populations were considered for inclusion in the MQTL analysis. Review articles, methodological papers, duplicate publications, and linkage mapping studies lacking sufficient marker information for projection onto the consensus genetic map were excluded. The five GWAS were retained separately for independent support of the identified MQTL. The literature search and study selection process are summarized in Figure 1, and the complete list of identified studies is provided in Table S1.

### 4.2. QTL Database Construction and Curation

For each eligible linkage mapping study, QTL information was extracted whenever available, including QTL designation, chromosome, mapping population, population size, parental lines, marker type, flanking markers, peak marker, genetic position (cM), logarithm of odds score, PVE, confidence interval, resistance trait, and publication source. To ensure consistency among studies, chromosome nomenclature, marker names, and genetic positions were standardized according to SoyBase. Missing marker information was retrieved from SoyBase whenever possible, whereas QTL lacking sufficient information for projection onto the consensus genetic map were excluded from the MQTL analysis. When multiple publications reported overlapping QTL derived from the same mapping population, duplicate entries were carefully evaluated and consolidated to retain the most complete and non-redundant dataset. Each retained QTL was assigned a unique study identifier to facilitate traceability throughout the meta-analysis workflow. Following marker verification, confidence interval estimation, and quality control, a final dataset comprising 153 unique SDS resistance QTL derived from 14 linkage mapping studies was assembled for consensus MQTL analysis. The complete curated QTL dataset used in this study is provided in Table S2.

### 4.3. QTL Standardization and Consensus MQTL Identification

All curated QTL were standardized prior to meta-analysis using a custom workflow implemented in R 4.6.0 (R Foundation for Statistical Computing, Vienna, Austria). Only QTL retained following literature curation, marker verification, and quality control were included in the analysis. Peak positions, flanking marker positions, confidence intervals, and PVE values were converted to numeric format to ensure consistency among studies. Rather than using conventional MQTL software packages, consensus MQTL were identified using a custom R-based workflow developed specifically for this study. This approach was designed to accommodate heterogeneous QTL datasets compiled from multiple independent studies while maintaining complete transparency and reproducibility. Published QTL information was standardized, confidence intervals and peak positions were harmonized, and overlapping QTL were grouped into consensus genomic regions using an interval-overlap clustering algorithm implemented independently for each chromosome. Consecutive QTL with overlapping confidence intervals were assigned to the same MQTL, whereas non-overlapping intervals initiated a new MQTL.

When peak positions were unavailable, they were estimated as the midpoint between the left and right flanking markers. Missing confidence interval boundaries were inferred from reported flanking marker positions whenever possible. If only the peak position was available and confidence interval boundaries could not be recovered from flanking-marker information, a provisional ±1 cM interval around the reported peak was used solely to permit interval-based clustering. This interval represents a computational assumption rather than an empirically estimated QTL confidence interval.

### 4.4. Identification and Characterization of Consensus MQTL

Each consensus region was assigned a unique MQTL identifier according to chromosome number and cluster order (e.g., MQTL18-1). For every MQTL, the chromosome, consensus confidence interval boundaries, number of supporting QTL, number of independent studies, supporting study identifiers, and maximum PVE were recorded. Consensus peak positions were estimated as PVE-weighted averages of the individual QTL peak positions, giving greater weight to QTL explaining larger proportions of the phenotypic variance. When PVE values were unavailable or equal to zero for all contributing QTL, the consensus peak position was calculated as the arithmetic mean of the individual peak positions. Only MQTL supported by two or more independent QTL were retained for subsequent physical localization, candidate gene identification, functional enrichment analysis, and integration with published GWAS.

### 4.5. Physical Localization of Consensus MQTL

Consensus MQTL were physically anchored to Wm82.a2.v1 by determining the physical positions of their peak markers using SoyBase. Candidate genes were identified by examining a 100-kb genomic interval (±50 kb) surrounding the physical position of each MQTL peak marker. This window was used as a consistent criterion to prioritize genes located near the consensus MQTL peak rather than to capture all genes within the complete MQTL confidence interval. All annotated genes located within these intervals were retrieved together with their corresponding gene identifiers, genomic coordinates, and functional annotations. Genes identified within overlapping search intervals were retained only once to generate a non-redundant candidate gene dataset for subsequent functional annotation, enrichment analysis, and GWAS comparison.

### 4.6. Candidate Gene Prioritization and Functional Annotation

Candidate genes identified within the MQTL intervals were compiled into a non-redundant dataset following the removal of duplicate gene models. Functional annotations, GO terms, and gene descriptions were retrieved from SoyBase. Candidate genes were manually classified according to their predicted biological functions, including disease resistance, signal transduction, transcriptional regulation, transport, hormone signaling, oxidative stress response, primary and secondary metabolism, protein degradation, and cellular development. Genes annotated as hypothetical, unknown, or uncharacterized proteins were retained in the complete candidate gene dataset but were not prioritized unless additional functional evidence was available. Well-supported candidate genes were prioritized based on their annotated or predicted involvement in plant immunity, defense responses, signal transduction, and biological processes previously associated with disease resistance in soybean and other plant species.

Functional enrichment analyses were performed using the complete non-redundant candidate gene dataset. GO enrichment analysis and KEGG pathway analysis were conducted using ShinyGO 0.81. GO biological processes and KEGG pathways with an FDR-adjusted *p*-value < 0.05 were considered significantly enriched.

### 4.7. GWAS Integration

To further evaluate the identified consensus MQTL, previously published GWAS investigating SDS resistance were systematically reviewed. Physical positions of significant SNPs reported in five independent GWAS were retrieved from the original publications and standardized according to Wm82.a2.v1 whenever necessary. Physical colocalization between significant GWAS SNPs and the identified MQTL was assessed by comparing the reported SNP positions with the physically mapped MQTL intervals. A GWAS SNP was considered colocalized when its physical position fell within the corresponding MQTL interval. Because the five GWAS were conducted using different germplasm populations with different linkage disequilibrium structures, a uniform LD-based window was not applied. MQTL containing one or more colocalized significant GWAS SNPs were considered supported by independent GWAS evidence.

### 4.8. Statistical Analysis and Data Visualization

All data processing, QTL standardization, consensus MQTL identification, candidate gene analyses, and functional enrichment analyses were performed using R. Data manipulation was conducted using the dplyr and readxl packages, and custom R scripts were developed to standardize QTL information, identify consensus MQTL, summarize chromosome-specific results, perform GO and KEGG enrichment analyses, and prepare datasets for downstream analyses. The study workflow (Figure 1) was created using Microsoft PowerPoint. Genome-wide visualization of curated QTL (Figure 2) and MQTL distribution (Figure 4) were generated using JMP 19 (SAS Institute Inc., Cary, NC, USA). Chromosome-level MQTL distribution (Figure 3), the physical colocalization of MQTL and published GWAS loci (Figure 5), and GO enrichment (Figure 6) were generated in RStudio 2026.05.1+225 (Posit Software, PBC, Boston, MA, USA) using the ggplot2 package. All analytical procedures were implemented using R scripts developed specifically for this study, ensuring complete traceability from the curated QTL database to the final MQTL, candidate gene, functional enrichment, and GWAS integration datasets.

## 5. Conclusions

This study presents a comprehensive meta-analysis of soybean SDS resistance by integrating published QTL, candidate gene identification, functional annotation, and independent GWAS support. A total of 153 curated QTL was consolidated into 23 consensus MQTL, substantially reducing redundancy among previously reported loci and identifying genomic regions associated with SDS resistance. Several MQTL, particularly those located on chromosomes 6, 8, 18, and 20, were supported by multiple independent studies and further supported through colocalization with published GWAS signals, highlighting their importance as well-supported resistance loci. Annotation-based candidate gene analysis identified numerous genes predicted to be associated with pathogen perception, signal transduction, transcriptional regulation, and secondary metabolism, while GO enrichment analysis identified response to salicylic acid as the only biological process that remained significant after FDR correction. These findings provide biological evidence supporting the identified MQTL and improve our understanding of the molecular mechanisms underlying quantitative resistance to SDS. The integrated genomic framework developed in this study provides a valuable resource for soybean genetics and improvement. The well-supported MQTL and prioritized candidate genes represent promising targets for marker-assisted selection, genomic selection, fine mapping, and functional validation. As additional genetic and genomic resources become available, integrating MQTL with transcriptomics, functional genomics, and genome-editing technologies will further accelerate the identification of causal genes and the development of soybean cultivars with durable resistance to SDS.

## Figures and Tables

**Table 1:** Characteristics of the QTL mapping studies included in the meta-analysis.

Reference	Year	Study Type	Mapping Population	Population Size	Resistance Source	Population Independence	No. of QTL Included
Collins et al.	2022	Linkage mapping	A: E09088 × E12901; B:E05226-T × E09014; C:E05226-T × E09088	269 (A-F4); 124 (B-F4); 226 (C-F4)	Multiple MSU-derived sources	Full	6
de Farias Neto et al.	2007	Linkage mapping + confirmation	A: Ripley × Spencer; B: PI 567374 × Omaha;	155 (A-F2); 91 (A-F5); 96 (B-F4); 163 (B-F8)	Ripley; PI 567374	Full	5
Anderson et al.	2015	Linkage mapping	MD96-5722 (Monocacy) × Spencer RILs	94 (F5:7)	MD96-5722/Monocacy	Full	19
Chang et al.	2020	Linkage mapping	PI 243518 × Sloan	400 (F2)	PI 243518	Full	1
Kazi et al.	2008	Linkage mapping	Flyer × Hartwig	92 (F5)	Hartwig	Full	2
Swaminathan et al.	2018	Linkage mapping	A: A95-684043 × LS94-3207; B: A95-684043 × LS98-0582	200 (A-F7); 200 (B-F7)	LS94-3207; LS98-0582	Full	6
Tan et al.	2018	Linkage mapping + epistasis	GD2422 × LD01-5907	129 (F4)	LD01-5907/Hartwig-derived	Full	4
Tan et al.	2019	Linkage mapping	U01-390489 × E07080	153 (F4)	E07080	Full	12
Abdelmajid et al.	2012	Linkage mapping	PI 438489B × Hamilton	50 (F6)	PI 438489B	Full	15
Iqbal et al.	2001	Linkage mapping	Essex × Forrest	100 (F5)	Forrest; Essex favorable alleles	Partial	6
Hnetkovsky et al.	1996	Linkage mapping	Essex × Forrest	100 (F5)	Forrest; Essex favorable alleles	Partial	2
Swaminathan et al.	2015	Linkage mapping with toxin assays	A: A95-684043 × LS94-3207; B: A95-684043 × LS98-0582	A: 200 (F7); B: 200 (F7)	LS94-3207; LS98-0582	Partial	15
Chang et al.	1996	Linkage mapping	Essex × Forrest	100 (F5)	Forrest	Partial	6

**Table 2:** Information of identified consensus meta-QTL (MQTL).

MQTL	Chr.	Consensus Confidence Interval (cM)	Consensus Peak (cM)	No. of Overlapping QTL	Supporting Studies	Maximum PVE (%)
MQTL1-1	1	15.4–16.1	15.7	2	1	0.9
MQTL1-2	1	22.4–42.8	35.0	3	3	7.5
MQTL2-1	2	19.4–21.8	20.6	2	2	9.0
MQTL2-2	2	30.0–36.0	33.0	2	2	5.2
MQTL3-2	3	15.7–16.1	15.8	2	1	0.8
MQTL3-3	3	28.1–49.4	40.5	5	3	9.9
MQTL4-2	4	51.5–83.9	62.7	5	3	14.0
MQTL5-1	5	8.5–11.7	9.5	2	1	0.0
MQTL6-2	6	97.8–201.0	140.9	9	4	24.1
MQTL6-1	6	0.0–74.1	29.0	11	6	12.4
MQTL8-1	8	2.0–13.0	7.5	2	2	9.6
MQTL8-2	8	15.0–103.5	33.3	11	5	17.4
MQTL9-2	9	45.7–51.5	48.6	2	2	13.0
MQTL10-2	10	13.5–15.2	14.4	2	2	19.3
MQTL11-1	11	5.5–17.8	11.7	2	2	3.4
MQTL13-1	13	0.0–5.8	2.7	2	2	0.1
MQTL14-2	14	10.3–18.2	12.0	2	1	6.4
MQTL15-1	15	1.4–3.0	2.5	2	1	0.6
MQTL17-1	17	3.2–47.9	17.0	2	2	7.5
MQTL18-3	18	66.0–76.1	71.0	2	1	--
MQTL18-1	18	0.0–39.3	14.6	13	6	33.3
MQTL19-2	19	42.0–49.9	46.0	2	2	17.7
MQTL20-1	20	2.8–113.8	46.0	9	5	15.0

**Table 3:** Prioritized candidate genes identified within consensus MQTL intervals.

Chr.	MQTL	Gene ID	Annotation	Functional Class	Putative Role in SDS Resistance
1	MQTL1-2	Glyma.01G163500	bZIP transcription factor	Transcription factor	Regulates defense- and stress-responsive gene expression.
2	MQTL2-1	Glyma.02G218400	MATE efflux family protein	Transporter	Mediates transport of defense-related metabolites and detoxification.
2	MQTL2-1	Glyma.02G226000	UDP-glycosyltransferase superfamily protein	Secondary metabolism	Participates in glycosylation of defense metabolites.
2	MQTL2-1	Glyma.02G226800	Phosphoinositide phospholipase C	Signal transduction	Mediates phospholipid-dependent immune signaling.
2	MQTL2-2	Glyma.02G245900	Calcium-binding EF-hand protein	Signal transduction	Regulates calcium-dependent defense signaling.
2	MQTL2-2	Glyma.02G265600	Thioredoxin family protein	Redox regulation	Maintains cellular redox homeostasis during pathogen infection.
2	MQTL2-2	Glyma.02G265900	Calcium-binding EF-hand protein	Signal transduction	Regulates calcium-dependent defense signaling
3	MQTL3-3	Glyma.03G227800	PIF3-like transcription factor	Transcription factor	Regulates stress-responsive signaling pathways.
3	MQTL3-3	Glyma.03G228300	Isopenicillin N epimerase-like protein	Defense metabolism	Participates in antimicrobial secondary metabolism.
4	MQTL4-2	Glyma.04G121100	Protein kinase family protein	Protein kinase	Activates defense signaling cascades.
4	MQTL4-2	Glyma.04G097000	SNARE family protein	Vesicle trafficking	Facilitates vesicle-mediated defense responses.
6	MQTL6-1	Glyma.06G260100	Disease resistance protein (TIR-NBS-LRR class)	Disease resistance protein	Recognizes pathogen effectors and activates immune responses.
6	MQTL6-2	Glyma.06G195100	Thioredoxin family protein 3	Redox regulation	Maintains ROS homeostasis during defense responses
8	MQTL8-2	Glyma.08G159200	Disease resistance protein (TIR-NBS-LRR class)	Disease resistance protein	Recognizes pathogen and activates immune responses
18	MQTL18-1	Glyma.18G039400	Protein kinase superfamily protein	Protein kinase	Mediates signal transduction during plant immune responses
18	MQTL18-3	Glyma.18G141500	Receptor-like kinase 1	Protein kinase	Regulates receptor-mediated defense signaling.
19	MQTL19-2	Glyma.19G155100	Cysteine-rich receptor-like protein kinase 10-like	Receptor-like kinase	Mediates pathogen perception and downstream signaling.
20	MQTL20-1	Glyma.20G020500	Disease resistance protein (TIR-NBS-LRR class)	Disease resistance protein	Recognizes pathogen and activates immune responses

**Table 4:** Genome-wide association studies (GWAS) used for independent support of meta-QTL (MQTL) for sudden death syndrome (SDS) resistance in soybean.

Reference	Year	Germplasm/Population	Population Size	SDS Trait(s)	Major Findings	Study Usage
Wen et al.	2014	A: Elite cultivars; B: advanced lines	392 (A); 300 (B)	Foliar SDS severity	Identified 20 loci associated with SDS resistance, including 13 novel loci, and refined the positions of previously reported resistance loci	Independent support of MQTL and candidate genes
Swaminathan et al.	2019	PI accessions	254	Foliar SDS severity	Detected significant SNPs within known SDS resistance loci and identified two putative novel genomic regions associated with SDS resistance	Independent support of MQTL
Rairdin et al.	2022	PI accessions, early maturity mini core	473	Foliar disease severity	Identified additive and epistatic loci associated with SDS resistance and proposed candidate defense-related genes	Comparison with MQTL intervals
Bao et al.	2015	Early-maturity soybean improvement lines	282	Foliar SDS severity	Identified novel SNP loci and candidate genes associated with foliar SDS and root rot resistance	Support of candidate genes
Zhang et al.	2015	PI accessions, germplasm accessions	214	Foliar SDS severity	Identified SNPs associated with SDS resistance, validating known loci and detecting additional candidate regions	Support of consensus MQTL

## Data Availability

The datasets generated and analyzed during this study are available within the article and its Supplementary Materials. Additional data used for the analyses are available from the author upon reasonable request.

## Supplementary Materials

The following supporting information can be downloaded at https://www.mdpi.com/article/10.3390/plants15172691/s1, Table S1, Complete literature survey of soybean sudden death syndrome (SDS) quantitative trait loci (QTL) mapping studies; Table S2, Curated dataset of 153 quantitative trait loci (QTL) used for consensus meta-QTL analysis.

## Abbreviations

The following abbreviations are used in this manuscript:

**Abbreviation**	**Definition**
SDS	Sudden death syndrome
QTL	Quantitative trait locus/loci
MQTL	Meta-QTL
GWAS	Genome-wide association study
SNP	Single nucleotide polymorphism
PVE	Phenotypic variance explained
CI	Confidence interval
LOD	Logarithm of odds
GO	Gene Ontology
KEGG	Kyoto Encyclopedia of Genes and Genomes
FDR	False discovery rate
RIL	Recombinant inbred line
SSR	Simple sequence repeat
RFLP	Restriction fragment length polymorphism
NLR	Nucleotide-binding leucine-rich repeat
